# A detailed characterization of complex networks using Information Theory

**DOI:** 10.1038/s41598-019-53167-5

**Published:** 2019-11-13

**Authors:** Cristopher G. S. Freitas, Andre L. L. Aquino, Heitor S. Ramos, Alejandro C. Frery, Osvaldo A. Rosso

**Affiliations:** 1 Instituto de Computação, Universidade Federal de Alagoas, Maceió, Brazil; 2 Instituto de Física, Universidade Federal de Alagoas, Maceió, Brazil; 3 Departamento de Ciência da Computação, Universidade Federal de Minas Gerais, Belo Horizonte, Brazil; 4 Instituto de Medicina Traslacional e Ingeniería Biomedica, Hospital Italiano de Buenos Aires & CONICET, Ciudad, Autónoma de Buenos Aires Argentina

**Keywords:** Computer science, Applied mathematics

## Abstract

Understanding the structure and the dynamics of networks is of paramount importance for many scientific fields that rely on network science. Complex network theory provides a variety of features that help in the evaluation of network behavior. However, such analysis can be confusing and misleading as there are many intrinsic properties for each network metric. Alternatively, Information Theory methods have gained the spotlight because of their ability to create a quantitative and robust characterization of such networks. In this work, we use two Information Theory quantifiers, namely Network Entropy and Network Fisher Information Measure, to analyzing those networks. Our approach detects non-trivial characteristics of complex networks such as the transition present in the Watts-Strogatz model from *k*-ring to random graphs; the phase transition from a disconnected to an almost surely connected network when we increase the linking probability of Erdős-Rényi model; distinct phases of scale-free networks when considering a non-linear preferential attachment, fitness, and aging features alongside the configuration model with a pure power-law degree distribution. Finally, we analyze the numerical results for real networks, contrasting our findings with traditional complex network methods. In conclusion, we present an efficient method that ignites the debate on network characterization.

## Introduction

Understanding how networks arrange their connections (structure), and how the information flows through their nodes (dynamics), is a breakthrough for many scientific fields that rely on network science to assess all kinds of phenomena. Recently, Information Theory methods gained the spotlight because of their ability to create a more quantitative and robust characterization of complex networks, as an alternative to traditional methods. Standard quantifiers such as Shannon Entropy and Statistical Complexity were adapted to network analysis, providing a different perspective when evaluating networks^1^.

The quantification of systems with multidimensional measures, in particular, a 2D representation or “plane representation” formed by Information Theory-based quantifiers is extensively applied to time series analysis and characterization. For instance, Rosso *et al*.^2^ employed the time causal Entropy-Complexity plane to distinguish stochastic from deterministic systems. The Entropy-Complexity plane is comprised of measures of entropy (${\mathcal H}$) and Statistical Complexity (${\mathscr{C}}$). Two pieces of information are required to calculate ${\mathscr{C}}$, namely the information content and the disequilibrium (${\mathscr{Q}}$) of the system. To evaluate the Statistical Complexity, we use the entropy as a measure of the information content, and the disequilibrium is expressed by the divergence between the current system state and an appropriate reference state. The calculation of these quantifiers requires the use of a proper probability distribution that represents the system under study. In the case of time series, the distribution derived from the symbolization proposed by Bandt and Pompe^3^ has been successfully used to capture the intrinsic time causality behavior of the underlying system (see Supplemental Material). The Shannon Entropy is a global disorder measure commonly used in many applications of the Information Theory field and the Entropy-Complexity plane. It is relatively insensitive to substantial changes in the distribution taking place in a small-sized region of the space. For these reasons, the Shannon Entropy is referred to as a global measure. The Statistical Complexity (${\mathscr{C}}$), when defined as a divergence in the space of entropies, is also a global measure. Alternatively, the Fisher Information Measure (${\mathcal F}$) can be interpreted as a measure of the ability to estimate a parameter, as the amount of information that can be extracted from a set of measurements, and also, a measure of the state of disorder of a system or phenomenon^4^. The Fisher Information Measure (${\mathcal F}$) is a local measure as it is based upon the gradient of the underlying distribution, being, thus, significantly sensitive to even tiny localized perturbations.

Recently, the Entropy-Complexity plane was extended and used in the context of complex networks. In ref. ^1^, the authors showed that networks of the same category tend to cluster into distinct regions. To calculate the two required quantifiers, ${\mathcal H}$, and ${\mathscr{C}}$, for complex networks, the authors used the probability distribution of a *random walker* traveling between two nodes to represent the topological properties of the network. Based on this distribution, they calculated the Shannon Entropy and the Statistical Complexity. For the evaluation of the disequilibrium, they used the Jensen-Shannon divergence between the actual network and *random* networks and used the last as a reference model. To obtain this divergence it is necessary to average several *random* networks with the same number of nodes, which is typically time-consuming. They demonstrated the applicability of their proposal to families of *Random* Erdős-Rényi^5^, *Small-World* Watts-Strogatz^6^, and *Scale-Free* Barabási-Albert^7^ networks. However, this plane presents several limitations, as regularly, the random networks overlap with all the other models, creating confusion and misleading the conclusions when evaluating the network’s features.

In this work, we propose the Fisher information quantifier, more sensitive to a local relationship between nodes, as a measure of network disorder. Alongside, we suggest the use of the Shannon-Fisher plane as an alternative to the Entropy-Complexity plane for network characterization. Our approach does not require the calculation of a divergence to a reference model, which decreases the computational burden. We analyze two different groups of networks: synthetic and real-world networks.

## Methods

### Network definition

We assume a graph *G*(**V**, **E**), where **V** is the set of nodes and **E** is the set of links (edges) as a suitable model of a network. The graph is represented by an adjacency matrix **A** with dimension *N* × *N*, *N* being the number of nodes in the network, where *a*_*ij*_ = 1 if a link exists between nodes *i* and *j*, otherwise, *a*_*ij*_ = 0. We consider undirected, unweighted, and without the presence of loops graph, i.e., simple unweighted graphs. Hence, their adjacency matrices have the main diagonal *a*_*ii*_ = 0, ∀*i* = 1, …, *N*, and **A** = **A**^*T*^. The node degree *k*_*i*_ is calculated by ${k}_{i}=\sum _{j}\,{a}_{ij}$, therefore, 0 ≤ *k*_*i*_ ≤ *N* − 1.

### Network entropy

Network Entropy is based on the classical Shannon Entropy for discrete distributions. Small^8^ proposed a measure of Network Entropy based on the probability that a random walker goes from node *i* to any other node *j*. This probability distribution *P*^(*i*)^ is defined for each node *i* and has entries

1 $${p}_{i\to j}=(\begin{array}{ll}0, & {\rm{for}}\,{a}_{ij}=0,\\ 1/{k}_{i}, & {\rm{for}}\,{a}_{ij}=1.\end{array}$$

It is easy to observe that ${\sum }_{j}{p}_{i\to j}=1$ for each node *i*.

Based on the probability distribution *P*^(*i*)^, the entropy for each node can be defined as

2 $${{\mathscr{S}}}^{(i)}\equiv {\mathscr{S}}[{P}^{(i)}]=-\,\mathop{\sum }\limits_{j=1}^{N-1}\,{p}_{i\to j}\,\mathrm{ln}\,{p}_{i\to j}=\,\mathrm{ln}\,{k}_{i}.$$

with ${{\mathscr{S}}}^{(i)}=0$ if node *i* is disconnnected.

After calculating the entropy for each node, we then calculate the normalized node entropy by

3 $${ {\mathcal H} }^{(i)}=\frac{{\mathscr{S}}[{P}^{(i)}]}{\mathrm{ln}(N-1)}=\frac{\mathrm{ln}\,{k}_{i}}{\mathrm{ln}(N-1)}.$$

Finally, the *normalized Network Entropy* is calculated averaging the normalized node entropy over the whole network as

4 $$ {\mathcal H} =\frac{1}{N}\mathop{\sum }\limits_{i=1}^{N}\,{ {\mathcal H} }^{(i)}=\frac{1}{N\,\mathrm{ln}(N-1)}\mathop{\sum }\limits_{i=1}^{N}\,\mathrm{ln}\,{k}_{i}.$$

The normalized Network Entropy is maximal ${\mathcal H} =1$ for fully connected networks, since *p*_*i* → *j*_ = (*N* − 1)^−1^ for every *i* ≠ *j* and the walk becomes fully random, i.e., jumps from node *i* any other node *j* are equiprobable. The walk becomes predictable in a sparse network because it limits the possibility of jumps. The sparser the network, the lower becomes its Network Entropy.

The normalized Network Entropy ${\mathcal H}$, hence, quantifies the heterogeneity of the network’s degree distribution, with lower values for nodes with lower degrees and higher values for nodes with higher degrees. For example, peripheral nodes present lower ${ {\mathcal H} }^{(i)}$ than hubs. Entropy, thus, ranges from ${\mathcal H} \to 0$ (sparse networks) to ${\mathcal H} \to 1$ (fully connected networks).

### Network fisher information measure

The *normalized Fisher Information Measure (FIM)*^9^ for a node *i* is given by

5 $${ {\mathcal F} }^{(i)}[{P}^{(i)}]=\frac{1}{2}\mathop{\sum }\limits_{j=1}^{N-1}\,{[\sqrt{{p}_{i\to j+1}}-\sqrt{{p}_{i\to j}}]}^{2}.$$

The normalized *network Fisher Information Measure* is given by

6 $$ {\mathcal F} =\frac{1}{N}\sum _{i}\,{ {\mathcal F} }^{(i)}[{P}^{(i)}].$$

If the system under study is in a very ordered state, i.e., a sparse network, almost all *p*_*i* → *j*_ values are zeros, we have Shannon Entropy ${\mathcal H} \to 0$ and normalized Fisher’s Information Measure ${\mathcal F} \to 1$. On the other hand, when a very disordered state represents the system under study, that is when all *p*_*i* → *j*_ values are similar, we obtain ${\mathcal H} \to 1$ and ${\mathcal F} \to 0$. We can then define a Shannon-Fisher plane, which can also be used to characterize Complex Networks.

## Results: Synthetic Networks

In this section, we analyze the behavior of Information Theory quantifiers when applied to Random (RN), Small World (SWN), and Scale-Free networks (SFN). We simulated independent instances of these networks for several parameters and then analyzed how their Network Entropy and Fisher Information Measure vary. These synthetic networks may present some degree of *stochasticity* related to its parameters setting, which results in variations of the quantifiers; for this, when we observe variations in any measure ${\mathscr{X}}$, we represent it by its average value $\bar{{\mathscr{X}}}$ along with its sampling standard deviation ${s}_{{\mathscr{X}}}$. These variations, when too small, can be hard to distinguish in figures, but their numerical results should make this clearer.

### Erdős-rényi: random networks

Boccaletti *et al*.^10^ state that: “*the term random graph refers to the disordered nature of the arrangement of links between different nodes*.” According to ref.^11^, Solomonoff and Rapoport^12^ initiated the study upon the nature of random graphs, but Erdős and Rényi^5^ are most known by observing the properties of networks as they increase the number of random connections, thus, defining an ensemble of graphs *G*_*N*,*M*_, with *N* nodes and *M* edges. Later, Gilbert^13^ described an alternative method for generating random graphs by defining an ensemble of graphs *G*_*N*,*p*_ with *N* nodes connecting randomly according to a *linking probability p* that is analogous to the *link density* $\xi ={(N(N-1))}^{-1}\,{\sum }_{i}^{N}{k}_{i}$. Although Erdős-Rényi (ER) random graphs are well studied in network science, they often fail at describing essential properties of real networks.

**Figure 1:**
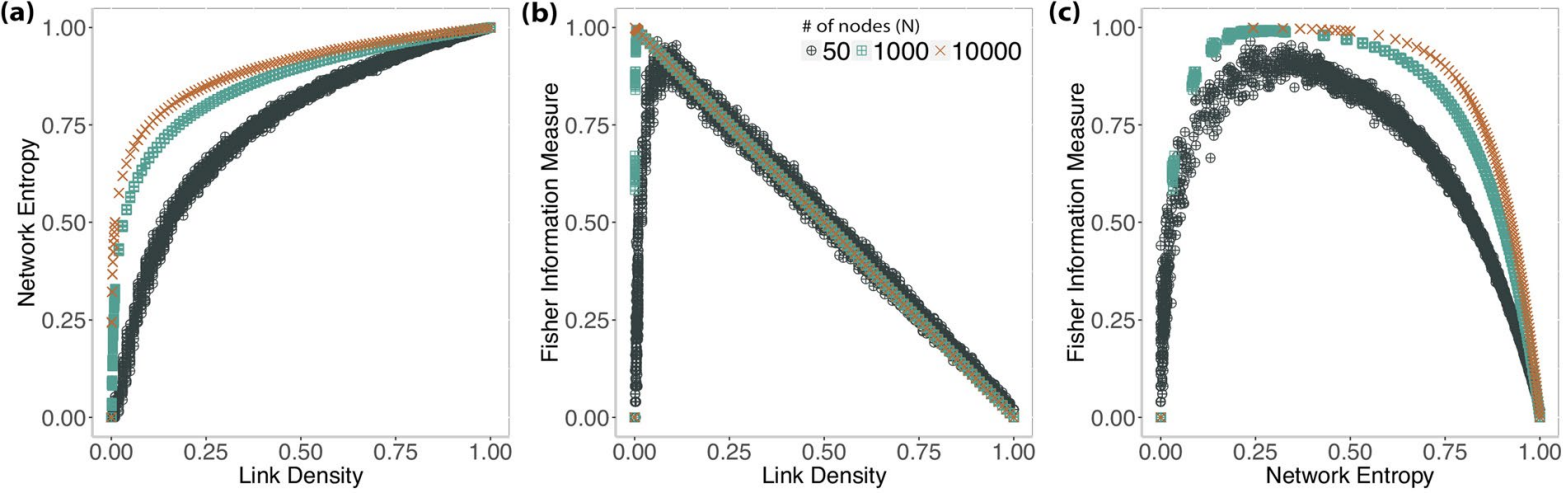
Results showing the relationship of Shannon Entropy and Fisher Information Measure with link density (**a**,**b**), and between Fisher Information Measure and Shannon Entropy (**c**) for 50 independent Erdős-Rényi networks. The dark-green circles corresponds to *N* = 50; the light-green squares to *N* = 1000; and the orange crosses to *N* = 10000.

We analyzed fifty independent ER graphs *G*_*N*,*p*_ for every combination of *N* = {50, 1000, 10000} and *p* ∈ {0, 0.001, 0.002, …, 0.99, 1} making, thus, a total of 50 × 3 × 1001 graphs. Figure 1 shows the variation of the Shannon Entropy (Fig. 1a) and Fisher Information Measure (Fig. 1b) with respect to the link density, while Fig. 1c depicts the relationship in between the Shannon Entropy and Fisher Information Measure.

Figure 1a shows how the Shannon Entropy ${\mathcal H}$ varies with respect to the link density *ξ*. The variation starts steep, then saturates. This may enhance the sensibility of Shannon-Fisher plane for sparse networks, but it may not be sensitive to denser graphs. The relationship between ${\mathcal H}$ and *ξ* also depends on the number of nodes *N*. The Shannon Entropy increases, for the same link density, with *N*. However, the rate of this growth decreases with *N*.

Figure 1b suggests that the Fisher Information Measure presents two distinct regimes for ER graphs as a function of their link density. Initially, this measure grows steadily: for *p* = 0 the network starts is totally disconnected; as *p* increases, it reaches a critical point *p*_*c*_ that is relative to the network number of nodes *N*, after which the measure decreases in a quasi-linear fashion ${\mathcal F} \approx 1-p$ for every *p* > *p*_*c*_, regardless *N*. For *N* = 50, the critical point is $\overline{{p}_{c}}=0.08$ with standard deviation *s*_*pc*_ = 0.02 and $\bar{ {\mathcal F} }=0.90$, ${s}_{ {\mathcal F} }=0.02$; for *N* = 1000, $\overline{{p}_{c}}=0.008$, *s*_*pc*_ = 0.002 with $\bar{ {\mathcal F} }=0.99$, ${s}_{ {\mathcal F} }=0.001$; for *N* = 10000, $\overline{{p}_{c}}=0.001$ with $\bar{ {\mathcal F} }=0.999$ and no variation observed. As the linking probability for ER graphs is analogous to the link density, this also stands for the link density, so $\bar{ {\mathcal F} }\approx 1-\xi$ for every *ξ* > *ξ*_*c*_. This result relates to the expected phase transitions in random graphs at *p*_*c*_ > ln*N*/*N*^14^, as the network will almost surely be connected.

Figure 1c shows the relationship between the Shannon Entropy and the Fisher Information Measure. As expected, the larger the network is, the less the variability observed. For this reason, we will only present results for *N* = 1000 hereinafter.

### Watts-strogatz: small-world networks

**Figure 2:**
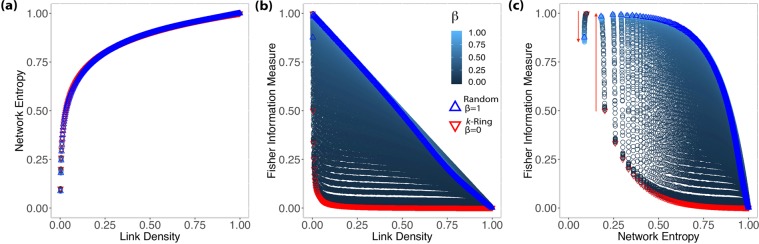
Results showing the relationship between Shannon Entropy and Fisher Information Measure with link density (**a**,**b**), and between Fisher Information Measure and Shannon Entropy for Watts-Strogatz networks (**c**). We restricted the analysis to *N* = 1000, $k\in \{1,2,3,\ldots ,499,500\}$ and $\beta \in \{0,0.001,0.002,\ldots ,0.99,1\}$; the downward red triangles correspond to $k$-rings (${G}_{N,k}$ with *β* = 0); the upwards blue triangles are random graphs (${G}_{N,k}$ with *β* = 1). The blue gradient from dark to light corresponds to rewiring probability *β*: the intensity of the blue color is inversely proportional to the value of *β*. The red arrows (**c**) identify the change of regime.

Small-world networks present an intrinsic characteristic of having relatively small average path length between nodes^15^. Watts and Strogatz^6^ (WS) proposed a model to build graphs *G*_*N*,*k*_ that can reproduce this *small-world* property with a high clustering coefficient. Start with a *k*-ring network with *N* nodes and a probability *β*. The rewiring consists of removing existing edges and connecting to another random node. When *β* = 0, we have a ring lattice, and for *β* = 1, it produces a random graph. For intermediate values, the model produces networks with the *small-world* property and a nontrivial clustering coefficient^10^. Figure 2 shows the same analysis as presented previously for random networks.

Figure 2a shows that the relationship between Network Entropy and link density is consistent with what was observed in Random Networks and that there is little variation with respect to *β*. Therefore, the Network Entropy ${\mathcal H}$ by itself does not provide information to identify different Small-World models.

Figure 2b shows the relationship between the Fisher measure and link density, indexed by the rewiring probability *β* (shades of blue). As expected, the behavior in the limit *β* = 1 is the same (linear decay) as the one observed for RN; cf. Figure 1b. There is a lower bound which corresponds to *k*-rings (red dots).

The red arrows (Fig. 2c) identify the change of regime. For *k* = 1 (red-downward arrow), increasing the rewiring probability decreases the Fisher ${\mathcal F}$ measure, while for *k* ≥ 2 (after red-upward arrow), this behavior is inverted, and increasing *β* also increases ${\mathcal F}$.

The Shannon-Fisher plane provides a rich description of Watts-Strogatz (WS) networks. Similarly to what was observed with ER graphs, there are two distinct regimes when evaluating WS graphs. Firstly, we see in Fig. 2 that for *k* = 1, we start with a ring lattice where ${\mathcal H} =0.1$, ${\mathcal F} =0.999$, and increasing *β*, we see ${\mathcal F}$ decreasing until it reaches a random graph; this happens because when *k* = 1 and *β* > 0, the rewiring mechanism isolates some nodes, and we have disconnected components lowering the ${\mathcal F}$ values. Secondly, for *k* > 1, WS has a different behavior, wherefore the rewiring mechanism most likely will not leave isolated nodes when *β* > 0; in fact, it will create larger components without fully disconnecting the other ones, this will increase the ${\mathcal F}$ values. The red arrows in Fig. 2 identify this change of regime. Alongside this, the Shannon-Fisher plane corroborates with the evidence of the transition between ring lattices and random graphs for the WS model.

**Figure 3:**
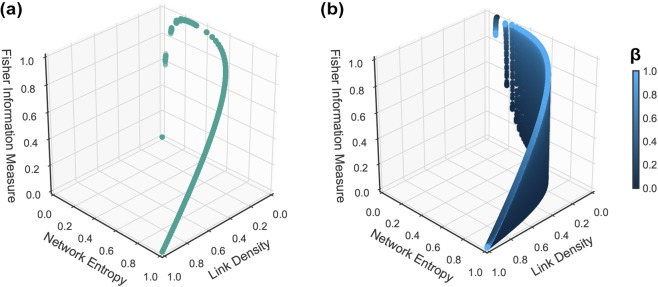
Relationship among the Network Entropy, Fisher Information Measure and link density for Erdős-Rényi (**a**) and Watts-Strogatz (**b**) networks in the ${\mathcal H} \times  {\mathcal F} \,\times \,\xi$ space.

Figure 3 summarizes the main differences between Erdős-Rényi (Fig. 3a) and Watts-Strogatz (Fig. 3b) networks in the ${\mathcal H} \times  {\mathcal F} \,\times \,\xi$ space. While Erdős-Rényi networks are equivalently well-described by the Network Entropy and the Fisher Information Measure (they span a 1D region of the space), Watts-Strogatz graphs are better characterized by the latter, as different networks span a 2D manifold. Permanent links to interactive versions of these 3D plots are available at http://tiny.cc/ERN and at http://tiny.cc/SWN.

### Barabási-albert: scale-free networks

The literature often uses *scale-free* networks as models for real networks. They have a degree distribution that can be fitted by a power-law, i.e., *P*(*k*) ~ *k*^−*γ*^, where *γ* is the degree exponent usually in 2 ≤ *γ* ≤ 3, as for *γ* > 3 the scale-free property can easily be confused with random networks^16^. We will evaluate the Barabási-Albert^7^ model (BA) for evolving *scale-free* networks, as it has two important features: network growth and the preferential attachment mechanism.

For network growth, at each time step *t*, new nodes are inserted with *m* links connecting with *N*_0_ existing nodes in the network. These links are created according to a probability given by the preferential attachment: the probability that a node *i* connects with *j* is proportional to the actual degree of node *i*:

7 $${\Pi }^{(i)}=\frac{{k}_{i}}{{\sum }_{j}\,{k}_{j}}.$$

In this way, the preferential attachment (PA) induces *hubs* (highly connected nodes), and peripheral communities, where nodes have similar degree. We know that the Barabási-Albert model is unable to reproduce all the diversity existing for scale-free networks, as it captures only the power-law with degree exponent *γ* = 3. Therefore, many variations of this model have been proposed throughout the years. In this work, we extend our analysis for: non-linear preferential attachment; the fitness property; the aging property; and finally, the configuration model.

#### Non-linear preferential attachment

Krapivsky *et al*.^17^ introduced a non-linear preferential attachment that creates different regimes for the network according to an exponent *α* controlling the network topology. The non-linear PA is given by

8 $${\Pi }^{(i)}=\frac{{k}_{i}^{\alpha }}{{\sum }_{j}\,{k}_{j}^{\alpha }}.$$

For *α* ≠ 1, the growth model stops resulting in a power-law degree distribution. There are, thus, three different growth regimes:

- The sublinear regime (*α* < 1) has a power-law with an exponential cutoff, where the preferential attachment is not strong enough to produce a pure power-law degree distribution.
- The linear regime (*α* = 1) has a pure power-law behavior corresponding to the *Barabási-Albert*^7^ model, with a resulting *γ* = 3.
- The superlinear regime (*α* > 1) presents a particular behavior where the network condensates, i.e, very few nodes win all connections; it also does not result in a power-law degree distribution.

**Figure 4:**
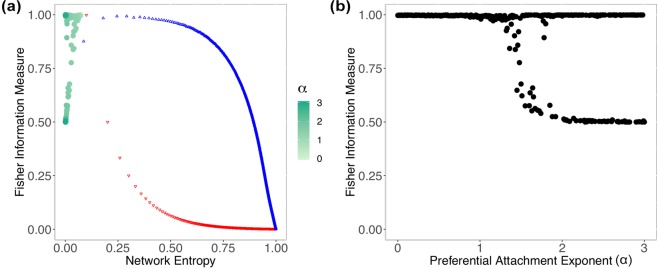
(**a**) Shows the results for Barabási-Albert networks using a non-linear preferential attachment with *N* = 1000 and $\alpha \in [0,3]$. For the sake of visualization, we plot the red downward triangles representing ${G}^{WS}$ with *β* = 0, i.e., *k*-ring graphs; and blue upward triangles representing ${G}^{WS}$ with *β* = 1, i.e., random graphs. (**b**) Shows how changing $\alpha$ causes disturbances in the Fisher Information Measure, when evaluating the Barabási-Albert model with non-linear PA.

We mapped outcomes of the BA model with a non-linear preferential attachment using the Krapivsky’s model onto the Shannon-Fisher plane, as shown in Fig. 4a. For *α* = 0, we have a random network, since Π(*i*) = 1 for every *i*, the network no longer obeys the preferential attachment mechanism, just the evolving growth property; the result is $\bar{ {\mathcal H} }=0.074$, ${s}_{ {\mathcal H} }=0.001$ and $\bar{ {\mathcal F} }=0.997$, ${s}_{ {\mathcal F} }=0.001$. Increasing *α* in steps of 0.01 changes the regime of the network slowly, and we see this transition in the Shannon-Fisher plane until it reaches *α* = 1. In the linear regime $\bar{ {\mathcal H} }=0.063$, ${s}_{ {\mathcal H} }=0.001$ and $\bar{ {\mathcal F} }=0.993$, ${s}_{ {\mathcal F} }=0.001$. In the superlinear regime ${\mathcal H} \to 0$ and ${\mathcal F}$ starts oscillating above *α* > 1.4, as seen in Fig. 4b. This oscillation happens due to the fact that after the network condensates, a small change in the network topology may cause ${\mathcal F}$ to drop from ${\mathcal F} =1$ to ${\mathcal F} =0.5$, as the Fisher Information Measure is sensitive to local disturbances.

**Figure 5:**
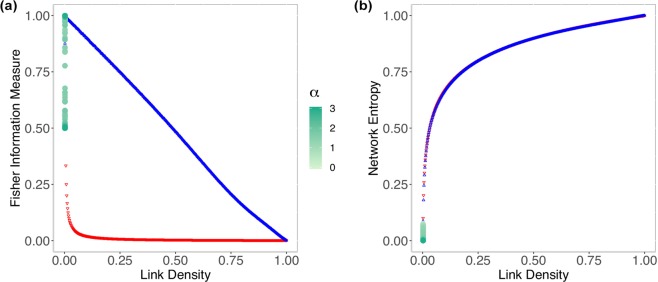
(**a**) Shows the relationship between link density and Fisher Information Measure for Barabási-Albert networks using a non-linear preferential attachment; the gradient indicates how the preferential attachment exponent *α* changes. (**b**) Shows the relationship between the Network Entropy and link density, where $\xi =0.002$ for any *α*. To help the visualization of the region where Barabási-Albert networks stand in relation to the other synthetic networks, we plot the red downward triangles representing ${G}^{WS}$ with *β* = 0, i.e., *k*-ring graphs, and blue upward triangles representing ${G}^{WS}$ with *β* = 1, i.e., random graphs.

Similar to earlier sections, we evaluated the link density *ξ* along with Network Entropy ${\mathcal H}$ and Fisher Information Measure ${\mathcal F}$. This time, the results with link density in comparison with Network Entropy, shown in Fig. 5b, have more interesting behavior. Although the link density does not change (*ξ* = 0.002), ${\mathcal H}$ absorbs the changes and when increasing *α*, ${\mathcal H} \to 0$. In Fig. 5a, we observe how Fisher Information Measure ${\mathcal F}$ against link density *ξ* produces confusing results, as the oscillation for *α* > 1.4 heads toward ${\mathcal F} \approx 0.5$ and ${\mathcal F} \approx 1$ with *ξ* = 0.002.

#### Fitness property

Some networks have nodes that create connections with more ability, e.g., a popular web page. Usually, these nodes gain relationships faster than common nodes. The Bianconi-Barabási model^18,19^ describes this property named *fitness*. We can model it using the preferential attachment considering a fitness coefficient *η*_*i*_ alongside the node degree *k*_*i*_:

9 $${\Pi }^{(i)}=\frac{{\eta }_{i}{k}_{i}}{{\sum }_{j}\,{\eta }_{j}{k}_{j}}.$$

In Eq. 9, the dependence of Π^(*i*)^ on *η*_*i*_ models the fact that even younger nodes can acquire links faster if they have sufficiently higher fitness than older nodes. Therefore, we draw 30 networks with *N* = 1000 considering a uniform distribution for the fitness *η*_*i*_ of each node *i*. For this, we do not expect a perfect power law, but we expect *γ* = 2.255, asymptotically.

**Figure 6:**
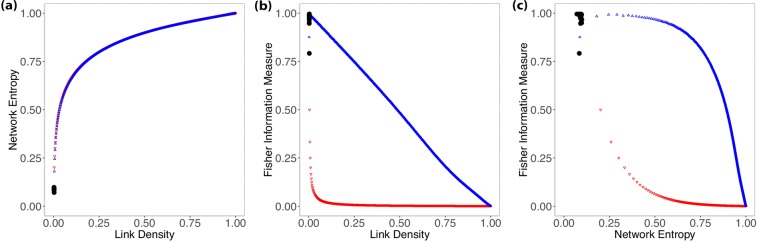
These results show the relationship between Shannon Entropy and Fisher Information Measure with link density (**a**,**b**), and between Fisher Information Measure and Shannon Entropy (**c**) for Biaconi-Barabási (Fitness model). Black points indicate the 30 networks with *N* = 1000 generated using a uniform distribution for the fitness scores of each node.

Figure 6a shows consistency between the Bianconi-Barabási, and the Barabási-Albert models, wherefore ${\mathcal H}$ grows slowly while *ξ* does not change and their numerical results are $\bar{{\rm{\xi }}}=0.00278$ with standard deviation *s*_*ξ*_ = 0.00006. Figure 6b also shows a similar behavior in comparison with Fig. 5a. Finally, Fig. 6c shows that most networks generated by the fitness model lie at a region close to the results presented for the BA model with $\bar{ {\mathcal H} }=0.091$, ${s}_{ {\mathcal H} }=0.005$ and $\bar{ {\mathcal F} }=0.979$, ${s}_{ {\mathcal F} }=0.038$.

#### Aging property

Another aspect we can also consider for a scale-free network is the *aging* property^20^. Regularly, for the Barabási-Albert model, we account only for the node degree or as seen before, the fitness coefficient. However, what happens when a node starts to reduce the rate of acquiring new links with time? This aging process causes the nodes to lose relevance; thus, it changes the network structure and dynamics. We can model this property considering:

10 $${\Pi }^{(i)}({k}_{i},t-{t}_{i}) \sim k{(t-{t}_{i})}^{-\nu },$$

where *ν* is a parameter controlling the dependence of the attachment probability on the node’s age. According to *ν*, we can define three scaling regimes:

- If *ν* < 0, new nodes will connect to older nodes. If *ν* → −∞, each new node connects to the oldest node, resulting in a condensed network or hub-and-spoke topology. Hence, we have a more heterogeneous network with a few hubs and many peripheral nodes.
- If *ν* > 0, nodes connect to younger nodes. By aging, nodes lose the ability of preferential attachment. In this case, the network tends to be more homogeneous.
- For *ν* > 1, the aging effect dominates the preferential attachment effect, the network loses its scale-free property, and it eventually approaches a random network. When *ν* → ∞, each node connects to its immediate predecessor.

**Figure 7:**
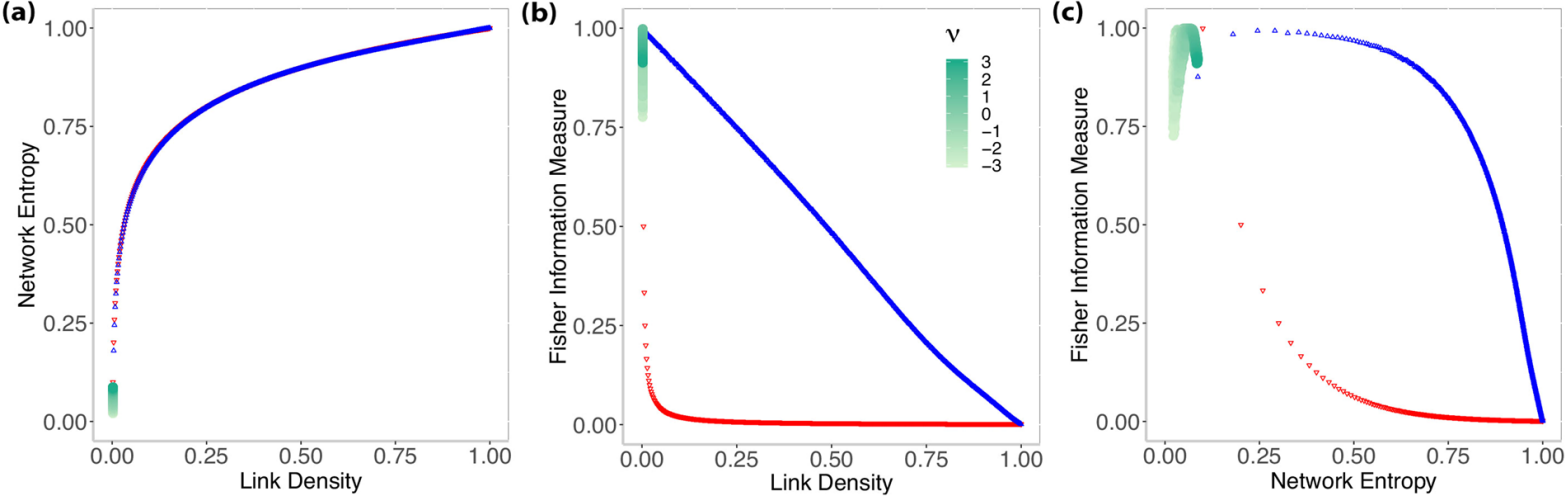
These results show the relationship between Shannon Entropy and Fisher Information Measure with link density (**a**,**b**), and between Fisher Information Measure and Shannon Entropy (**c**) for the Aging model. The gradient indicates the aging exponent $\nu \in [\,-\,3,3]$ and how its growth controls the network scaling regimes.

For evaluating the aging property, we generate distinct networks with *N* = 1000 and *ν* ∈ {−3.0, −2.9, −2.8, ..., 2.9, 3.0} with 30 replications of each setting; thus, we have a total of 18030 networks. Figure 7a shows the results for networks with a growing Network Entropy ${\mathcal H}$ and a steady link density *ξ* = 0.002, the same result as for BA model. Figure 7b shows the results for the aging property, and once more, we can observe the “oscillation” that happens to all the other scale-free models previously discussed. Figure 7c presents the results considering the Network Entropy ${\mathcal H}$ and Fisher Information Measure ${\mathcal F}$, where we can see the Aging model transition in the plane according to its scaling regimes.

The numerical results for the Aging model are the following: for *ν* = −3, $\bar{ {\mathcal H} }=0.024$, ${s}_{ {\mathcal H} }=0.0014$ and $\bar{ {\mathcal F} }=0.845$, ${s}_{ {\mathcal F} }=0.042$ i.e., we have a condensed network; when *ν* > 0, ${\mathcal H}$ and ${\mathcal F}$ continue to grow until *ν* = 1, wherefore $\bar{ {\mathcal H} }=0.072$, ${s}_{ {\mathcal H} }=0.0009$ and $\bar{ {\mathcal F} }=0.992$, ${s}_{ {\mathcal F} }=0.0021$; for *ν* > 1, ${\mathcal H}$ grows steadily and ${\mathcal F}$ decays, reaching a random regime. Finally, we noticed that the scale-free regime expected for *ν* ∈ [0, 1] is observed in the Shannon-Fisher plane, where the values for $\bar{ {\mathcal H} }=0.063$, ${s}_{ {\mathcal H} }=0.005$ and $\bar{ {\mathcal F} }=0.990$, ${s}_{ {\mathcal F} }=0.0064$.

#### The configuration model

A recurrent problem is “how do we generate networks with an arbitrary *P*(*k*)?”. For this, we use the configuration model, also known as a random network with a pre-defined degree sequence^21^. According to ref.^16^, the algorithm consists of the following steps:

1. Assign a degree to each node as stubs or half-links. It is required that we start from an even number of stubs; otherwise, we will have unpaired stubs.
2. Randomly selects a pair of half-links and connect them; then randomly choose another pair from the remaining 2*L* − 2 half-links and connect them.
3. Repeat this process until all stubs are paired up. Depending on how we pair them up, we may obtain distinct networks. Some networks include cycles, self-loops, or multi-links. In this work, we consider only simple graphs, thus, after generating the network for a degree sequence, we “simplify” the graph, removing self-loops and multi-links.

As we are trying to reproduce scale-free properties using the configuration model, we assign a pure power-law distribution *P*(*k*) = *k*^−*γ*^ with *γ* ∈ [2, 5]. For this model, we expect that for 2 ≤ *γ* ≤ 3, the network is in the scale-free regime; when *γ* > 3, the network starts to condensate, as the distribution has a steep curve. It means that few nodes have most of the links and most nodes have few links. Such networks present structure and dynamics more similar to a hub-and-spoke topology.

**Figure 8:**
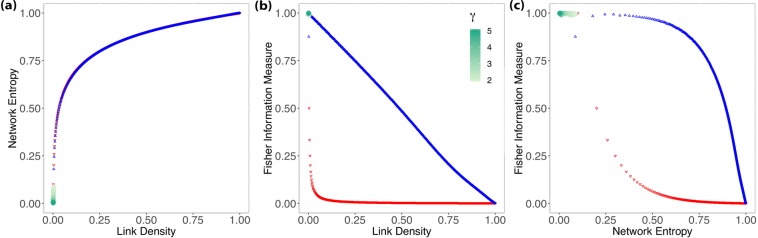
These results show the relationship of Shannon Entropy and Fisher Information Measure with link density (**a**,**b**), and between Fisher Information Measure and Shannon Entropy (**c**) for the configuration model with a degree distribution following a pure power-law $P(k) \sim {k}^{-\gamma }$. The gradient indicates the degree exponent $\gamma \in [2,5]$ and how it controls the network scaling regimes.

Finally, we evaluate these networks with *N* = 1000 using a pure power-law distribution with *γ* ∈ {2.0, 2.1, 2.2, ..., 4.9, 5.0}; as we cannot guarantee that networks with the same degree exponent have the same topology, we replicate this experiment 30 times, then, we have 1312 networks. In Fig. 8a we have that $\bar{{\rm{\xi }}}=0.001$, with *s*_*ξ*_ = 0.0006 while the Network Entropy ${\mathcal H}$ decreases as we increase the degree exponent. This behavior is similar for all the other scale-free models when we are in the condensed regime. Figure 8b shows how the ${\mathcal F}$ values are stable for this model with $\bar{ {\mathcal F} }=0.998$, ${s}_{ {\mathcal F} }=0.001$. And later, in Fig. 8c, we observe how Network Entropy ${\mathcal H}$ is actually the one capturing the changes, wherefore the degree exponent *γ* ∈ [2, 3] we have $\bar{ {\mathcal H} }=0.045$, ${s}_{ {\mathcal H} }=0.017$ and $\bar{ {\mathcal F} }=0.998$, ${s}_{ {\mathcal F} }=0.001$; and for *γ* ∈ [3, 5], we have $\bar{ {\mathcal H} }=0.010$, ${s}_{ {\mathcal H} }=0.005$ and $\bar{ {\mathcal F} }=0.998$, ${s}_{ {\mathcal F} }=0.001$.

**Figure 9:**
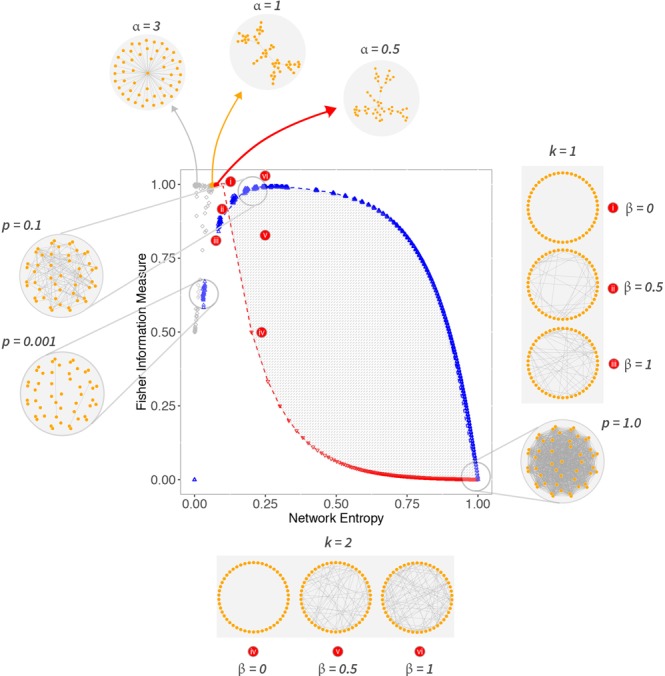
An overview of the most significant results for network models discussed in earlier sections. All the topologies presented have *N* = 50, and they are only a guide to understanding our proposal. Networks with labels $p$ indicate ER networks; WS graphs are indexed by *β* and *k*; and *α* is the parameter for networks with a nonlinear preferential attachment model; other network growth models discussed in this work (e.g., fitness model) fall together with the Barabási-Albert model with nonlinear preferential attachment.

Figure 9 summarizes the main features observed in the Shannon-Fisher plane for simulated networks, alongside examples illustrating different topologies and their expected results in the Shannon-Fisher plane. The *fitness*, *aging*, and *configuration* models were left out of this plot, as they are represented well enough by the Barabási-Albert model with nonlinear preferential attachment and its distinct scaling regimes. Considering the standard definition for the scale-free property and the network models evaluated, we observe how scale-free networks are subtle and can be easily confused with others in the Shannon-Fisher plane, as the usual result for scale-free networks is confined to a tiny region of the plane, and a few inputs can dismantle the scale-free property. This observation reflects a recent discovery that states that “scale-free networks are rare”^22^.

## Results: Real Networks

We evaluate real networks, assessing their topological features such as clustering coefficient *C*^Δ^, average path length *L*, and if the degree distribution follows a power law (*P*(*k*) ~ *k*^−*γ*^); we also consider the degree exponent *γ*. Table 1 shows the real networks analyzed in this work. Each network is presented with its number of nodes *N*, average degree 〈*k*〉, and link density *ξ*. We inform their small-world indicators: average path length *L*, clustering coefficient *C*^Δ^, small-world-ness value *S*^Δ^ (see below). We also provide scale-free indicators: degree exponent *γ* and *p*-value for the power-law fitting. Finally, we provide the Network Entropy ${\mathcal H}$ and Fisher Information Measure ${\mathcal F}$, along with a literature reference.

**Table 1:** Real networks and their descriptors.

ID	Network	*N*	〈*k*〉	*ξ*	*L*	*C*^Δ^	*S*^Δ^	*γ*	*p*-value	ℋ	ℱ	ref.
1	Email Network	1133	9.622	0.009	3.606	0.166	17.146	6.775	1.000	0.253	0.925	^26^
2	Adolescent Health	2539	8.236	0.003	4.559	0.142	38.118	8.244	0.996	0.248	0.902	^27^
3	Arxiv AstroPh	18772	21.101	0.001	4.194	0.318	239.693	4.496	0.980	0.231	0.992	^28^
4	NetScience Collaborations	1461	3.754	0.003	5.823	0.693	271.946	3.607	0.401	0.141	0.696	^29^
5	Science Collaborations	23133	8.078	0.000	5.352	0.264	737.276	3.426	0.310	0.160	0.996	^16^
6	Slucene	2956	7.336	0.002	4.499	0.057	22.710	2.187	0.896	0.174	0.894	^30^
7	AS Caida	16301	4.043	0.000	3.771	0.008	66.782	2.124	1.000	0.070	0.995	^31^
8	Power Grid	4941	2.669	0.001	18.989	0.103	92.461	7.629	1.000	0.094	0.967	^6^
9	Amazon pages	2879	2.700	0.001	3.433	0.023	56.123	3.257	0.000	0.016	0.956	^32^
10	Roget’s Thesaurus	1010	7.224	0.007	4.075	0.134	17.770	6.246	0.919	0.250	0.927	^33^
11	Autobahn	1168	4.257	0.002	19.419	0.003	0.745	7.050	0.000	0.099	0.998	^34^
12	Protein Interactions	2018	2.681	0.001	5.611	0.024	23.907	2.782	1.000	0.077	0.971	^16^
13	Drosophila Medulla 1	1781	10.007	0.006	2.911	0.069	14.828	3.957	0.986	0.198	0.982	^35^
14	Mouse Retina 1	1076	168.794	0.157	1.861	0.400	2.526	2.312	0.000	0.693	0.725	^36^

**Figure 10:**
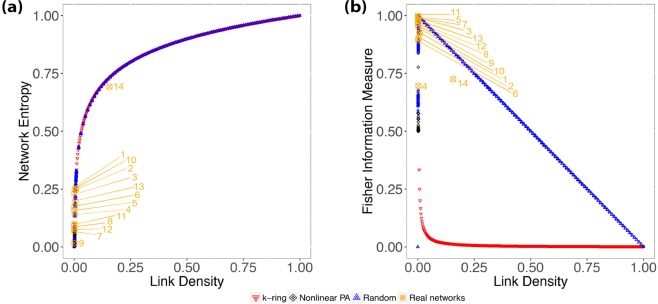
(**a**) Shows the relationship between link density and Network Entropy for real networks (Table 1), while (**b**) shows the relationship between link density and Fisher Information Measure. For the sake of visualization, we plot the red downward triangles representing ${G}^{WS}$ with *β* = 0, i.e., *k*-ring graphs, and blue upward triangles representing ${G}^{WS}$ with *β* = 1, i.e., random graphs.

As shown in Fig. 10a,b, most real networks are *sparse*, with *ξ* ≤ 0.009. The only exception is network #14 with *ξ* = 0.157. Therefore, our analysis will rely on our ability to distinguish sparse networks, with link density *ξ* that does not present distortions in Network Entropy ${\mathcal H}$ (Fig. 1a) nor Fisher Information Measure ${\mathcal F}$ (Fig. 1b). Nevertheless, care is needed not to jump into conclusions without further analysis, as differences in the Shannon-Fisher plane for sparse networks are subtle; thus, it is where other metrics are welcome in helping to confirm our findings.

Foremost, considering the results for the Watts-Strogatz model in the Shannon-Fisher plane, it is expected that networks in between the upper (i.e., random) and lower (*k*-ring) limits are very likely to be *small-world* networks. Although, this should not be confused with saying that these networks have the same topology. Our purpose here is to study how the information flows through the nodes. Networks may present similar topology that will result in similar dynamics, but distinct topologies may have similar dynamics; this feature is noteworthy.

**Figure 11:**
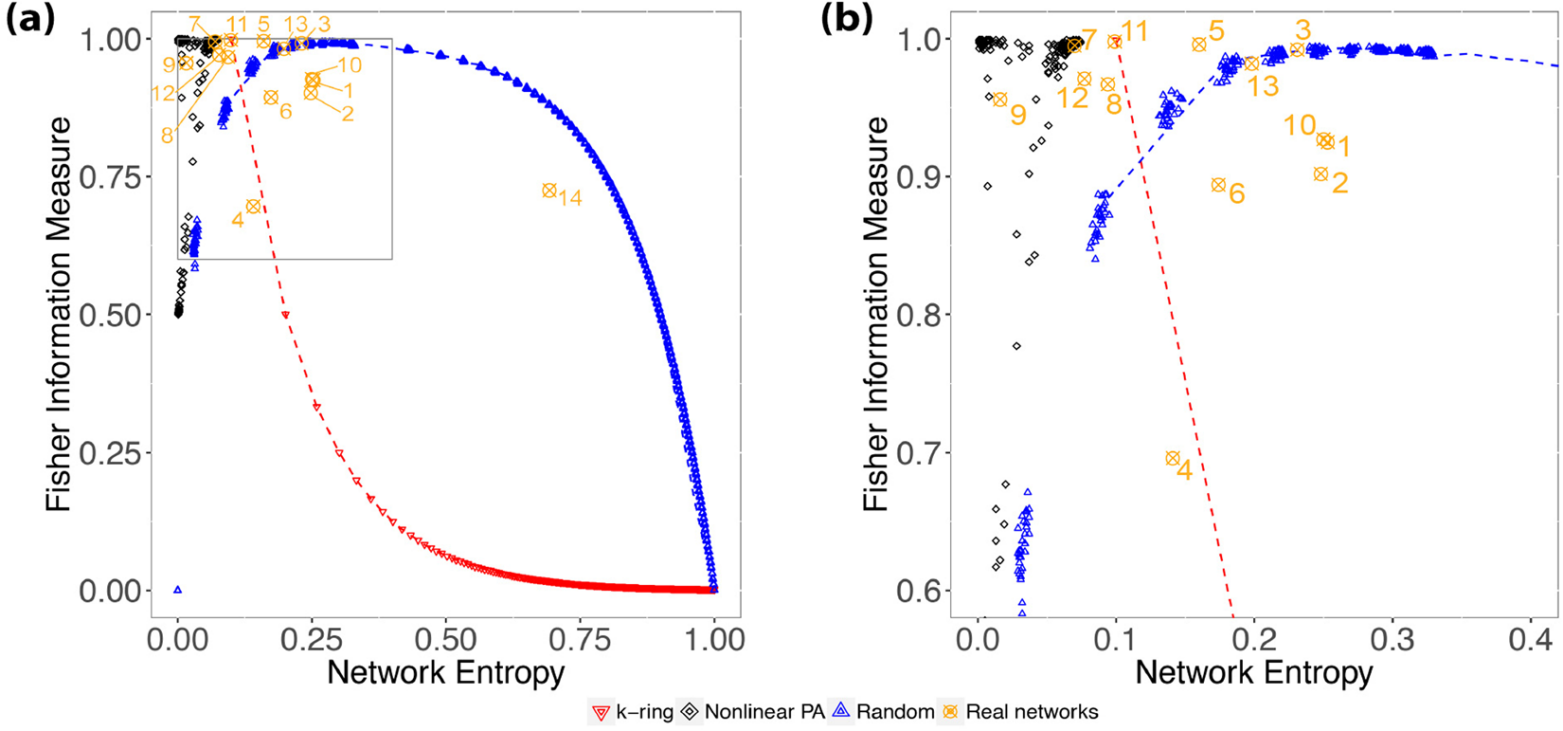
(**a**) Shows the relationship between Fisher Information Measure and Shannon Entropy for the real world networks. Blue upward triangles represent the ER graphs; the dashed-blue line indicates the upper limit of the small-world region delimited by graphs ${G}^{WS}$ with *β* = 1; the downward red triangles represent *k*-ring graphs, as the dashed-red line indicates a “rough” lower limit for the small-world region. (**b**) Zooms over the black points which represent the networks generated using a non-linear preferential attachment mechanism.

From the results in Fig. 11a and in Table 1, we may state that networks #1, #2, #4, #6, #10, #13 and #14 present *small-world* behavior. That said, we observe that the average path length *L* for these networks in Table 1 is small, considering its number of nodes *N*. Cohen and Havlin^23^ demonstrated that WS networks under some *expected conditions* have an average path length that scales as log*N*, and we can see that the largest network in our small-world set has *N* = 4941, therefore, as log*N* ≈ 8.50, and observing that *L* for every small-world network in our study has *L* < 8.5, following this criterion, they present the small-world behavior.

Another aspect that we can observe for small-world networks is their clustering coefficient (*C*^Δ^), but there is no certainty of which values to expect. In an attempt to perform quantitative analysis for clustering coefficient in small-world networks, and considering a relationship with the average path length, Humphries *et al*.^24^ proposed the small-world-ness *S*^Δ^, defined as

11 $${S}^{\Delta }=\frac{{C}^{\Delta }/{C}_{{\rm{rand}}}^{\Delta }}{L/{L}_{{\rm{rand}}}},$$

where *C*^Δ^ and *L* are, respectively, the clustering coefficient and average path length, and *C*_rand_^Δ^ and *L*_rand_ are the results computed for an ensemble of 100 ER networks, simulated with the same link density *ξ* as the real network. With this approach, Humphries *et al*.^24^ state that for *S*^Δ^ > 1, the network can be considered small-world.

However, Table 1 shows that only network #11 has *S*^Δ^ < 1, and if we analyze only the small-world-ness value, we may attest that all of the other networks are also small-world. This is inaccurate, if not wrong.

Alongside the small-world-ness value, as an attempt to identify scale-free networks, we can also estimate the degree exponent *γ* of the power-law degree distribution. Using a method proposed by Newman^25^, we reject the fit whenever *p*-value < 0.05, and if the estimated *γ* lies between two and three (2 < *γ* < 3), we consider these networks *scale-free*; for *γ* > 3, these networks may present hubs, but they become hard to distinguish from random or small-world networks.

We proceed with our study zooming into the Shannon-Fisher plane, as we can see in Fig. 11b. First, we observe networks #5 and #8, which are outside the small-world region, and network #3 that is overlapping the upper limit. We state that these three networks are *random*; Table 1 shows that they have *γ* > 3 with *p*-value > 0.05.

Although the identified random networks are outside the small-world region, they could easily be confused with scale-free or even regular networks considering just the results provided by the Shannon-Fisher plane, therefore, we ought to be careful with networks outside the small-world region, and for these cases, we must have a look into the degree distribution and estimate the degree exponent.

Network #7 is mapped into the ${\mathcal H} \times  {\mathcal F}$ plane closely to the classic Barabási-Albert model, along with *γ* = 2.124 and *p*-value = 1, thus, we cannot reject the fitting for a power-law, and we expect this network to be scale-free. Also, network #12 has *γ* = 2.782 with *p*-value = 1, and it is also close to network #7, although it is even closer to network #8.

Another “odd” result is the fact that network #6, although its results and properties signal for a small-world network, has degree exponent *γ* = 2.187 with *p*-value = 0.896, also indicating the scale-free property. Finally, network #9 has resulted in a point in ${\mathcal H} \times  {\mathcal F}$ equal to those generated with *superlinear* preferential attachment, leading to a “condensed” network. Indeed, with *L* = 3.771 and a degree distribution that does not fit a power-law at all, we state this network is “condensed.”

## Discussion

Complex networks have many faces, thus attempting to label them considering a single network property may be misleading. Real networks have many components and distinct interactions among them, for example, a scale-free network may have peripheral communities that lead to small-world structure. Our proposal quantifies network structure and dynamics, considering a simplified plot. We show consistent results with other network features when this methodology is applied to synthetic networks.

The Shannon-Fisher plane enhances our ability to evaluate complex networks:

- The transition that the Watts-Strogatz model exhibits in between k-ring and random graphs, leading us to define the small-world region;
- The two distinct regimes for the Erdős-Rényi model when reaching the critical linking probability;
- The three regimes for the non-linear preferential attachment on scale-free networks and distinct growth models, which transits between random, scale-free and condensed networks;
- The behavior of the fitness model when we consider a uniform distribution for the fitness of each node, and how it has similar features to the Barabási-Albert model;
- The effect of aging for scale-free networks and how the aging exponent can control the system’s behavior in the same manner to what happens with the non-linear preferential attachment;
- And finally, how we can generate networks with a pure power-law considering distinct degree exponent.

The evaluation of real networks gave us a peek into the real world and its deceitful aspects. Our method succeeds in characterizing most of the real networks in comparison with synthetic networks, even though a few examples showed unexpected behaviors that will be widely explored further. That said, our proposal is not the perfect fit for labeling networks as a small-world or scale-free, but it opens a world of possibilities when evaluating information spread, network robustness, or controllability. Our approach allows identifying distinct interactions in real networks, observing how they transit within the Shannon-Fisher plane and comparing how they affect other network features.

## Supplementary information

Supplemental Material

## Data Availability

The datasets generated during and/or analysed during the current study are available in the *fisher-networks* repository, https://gitlab.com/cristophersfr/fisher-networks.
