# Author Correction: A detailed characterization of complex networks using Information Theory

**DOI:** 10.1038/s41598-021-81323-3

**Published:** 2021-01-12

**Authors:** Cristopher G. S. Freitas, Andre L. L. Aquino, Heitor S. Ramos, Alejandro C. Frery, Osvaldo A. Rosso

**Affiliations:** 1 Instituto de Computação, Universidade Federal de Alagoas, Maceió, Brazil; 2 Instituto de Física, Universidade Federal de Alagoas, Maceió, Brazil; 3 Departamento de Ciência da Computação, Universidade Federal de Minas Gerais, Belo Horizonte, Brazil; 4 Instituto de Medicina Traslacional E Ingeniería Biomedica, Hospital Italiano de Buenos Aires & CONICET, Ciudad, Autónoma de Buenos Aires, Argentina

Correction to *Scientific Reports* 10.1038/s41598-019-53167-5, published online 13 November 2019

This Article contains errors.

After publication of the Article it was brought to Authors' attention that since the quantifier they were proposing for networks consider the node indices to build the random-walk-based distribution, if these indices are permuted without altering the network structure, the results for Network Fisher may change. This is a limitation of the original study, which should be acknowledged.

In order to overcome this limitation, we recommend using a matrix reordering algorithm, e.g., Optimal Leaf Ordering, that enhances the presence of block-patterns in the matrix, allowing the quantifier to characterize the network structure based on the adjacency matrix representation. For more information, readers can refer to^1^.

These limitations do not affect the overall conclusions of the Article.
