# Supplementary material for: A detailed characterization of complex networks using Information Theory
Source: Sci Rep. 2019 Nov 13;9:16689. doi: 10.1038/s41598-019-53167-5 (PMC6853913; doi:10.1038/s41598-019-53167-5)
Supplement: Supplementary file 1 — Supplemental Material [file 41598_2019_53167_MOESM1_ESM.pdf]

A detailed characterization of complex networks using Information Theory

Cristopher G. S. Freitas[1,*], Andre L. L. Aquino[1], Heitor S. Ramos[3], Alejandro C. Frery[1], and Osvaldo A. Rosso[2,4]

[1]Instituto de Computação, Universidade Federal de Alagoas, Maceió, Brasil
[2]Instituto de Física, Universidade Federal de Alagoas, Maceió, Brasil
[3]Departamento de Ciência da Computação, Universidade Federal de Minas Gerais, Belo Horizonte, Brasil
[4]Instituto de Medicina Traslacional e Ingeniería Biomedica, Hospital Italiano de Buenos Aires & CONICET, Ciudad Autónoma de Buenos Aires, Argentina.
[*]cristopher@laccan.ufal.br

Appendix A Shannon Entropy, Fisher Information Measure, and Statistical Complexity

An Information Theory quantifier is a measure able to characterize some property of the probability distribution function (PDF) associated with a phenomenon or a model. Entropy, regarded as a measure of uncertainty, is the most paradigmatic example of these quantifiers.

Given a continuous probability distribution function (PDF) $f(x)$ with $x \in \Omega \subset \mathbb{R}$ and $\int_\Omega f(x)\ dx = 1$, its associated *Shannon Entropy* (or Shannon's logarithmic information measure) $\mathcal{S}$ [11, 12] is defined by

$$\mathcal{S}[f] = -\int_\Omega f(x) \ln f(x) dx. \tag{1}$$

In the discrete case the PDF is given by $P \equiv \{p_j; j = 1, \ldots, N\}$ with $\sum_{j=1}^{N} p_j = 1$, with N the number of possible states of the system under study. The Shannon Entropy is defined by

$$\mathcal{S}[P] = -\sum_{j=1}^{N} p_j \ln p_j. \tag{2}$$

This functional equals zero when we are able to predict with full certainty which of the possible outcomes j will actually take place. Our knowledge of the underlying process, described by the probability distribution, is maximal in this instance. In contrast, this knowledge is commonly minimal for a uniform distribution $P_e = \{p_j = 1/N, \forall j = 1, \ldots, N\}$.

We define a "normalized" Shannon Entropy, $0 \le \mathcal{H} \le 1$, as

$$\mathcal{H}[P] = \mathcal{S}[P]/\mathcal{S}[P_e] = \mathcal{S}[P]/\ln N. \tag{3}$$

The Shannon Entropy $\mathcal{S}$ is a measure of "global character" not too sensitive to strong changes in the PDF taking place in small regions. This is not the case for the Fisher information measure [9, 2]

$$\mathcal{F}[f] = \int \frac{|\vec{\nabla} f(x)|^2}{f(x)} dx, \tag{4}$$

which constitutes a measure of the gradient content of the distribution f (continuous PDF), thus being quite sensitive even to tiny localized perturbations.

Local sensitivity is useful in scenarios whose description necessitates an appeal to a notion of "order". The concomitant problem of loss of information due to the discretization has been thoroughly studied (see, for instance, [13, 8] and references therein) and, in particular, it entails the loss of Fisher's shift-invariance, which is of no importance for our present purposes. For Fisher information measure computation (discrete PDF) we follow the proposal of Dehesa and coworkers [10] based on the amplitude of probability $f(x) = \psi(x)^2$ then

$$\mathcal{F}[\psi] = 4 \int \left\{ \frac{d\psi}{dx} \right\}^2 dx. \tag{5}$$

Its discrete normalized version ($0 \leq \mathcal{F} \leq 1$) is now

$$\mathcal{F}[P] = F_0 \sum_{i=1}^{N-1} (\sqrt{p_{i+1}} - \sqrt{p_i})^2. \tag{6}$$

Here the normalization constant F_0 reads

$$F_0 = \begin{cases} 1 & \text{if } p_{i^*} = 1 \text{ for } i^* = 1 \text{ or } i^* = N \text{ and } p_i = 0, \forall i \neq i^*, \\ 1/2 & \text{otherwise.} \end{cases} \tag{7}$$

If our system lies in a very ordered state, we can consider it is described by a PDF given by $P_0 = \{p_k \cong 1; p_i \cong 0, \forall i \neq k; i = 1, \ldots, N\}$ (with N, the number of states of the system) in consequence we have a Shannon Entropy $\mathcal{S}[P_0] \cong 0$ and a normalized Fisher's information measure $\mathcal{F}[P_0] \cong F_{\max} = 1$. On the other hand, when the system under study is represented by a very disordered state, one can think this particular state is described by a PDF given by the uniform distribution $P_e = \{p_i = 1/N, \forall i = 1, \ldots, N\}$ and we obtain $\mathcal{S}[P_e] \cong S_{\max}$ while $\mathcal{F}[P_e] \cong 0$. One can state that the general behavior of the Fisher information measure is opposite to that of the Shannon Entropy.

Complexity denotes a state of affairs that one can easily appreciate when confronted with it; however, it is rather difficult to define it quantitatively, probably because there is no universal definition of complexity. In between the two special instances of perfect order and complete randomness, a wide range of possible degrees of physical structure exists that should be reflected in the features of the underlying probability distribution P. One would like to assume that the degree of correlational structures would be adequately captured by some functional $\mathcal{C}[P]$ in the same way that Shannon's entropy $\mathcal{S}[P]$ captures randomness [11].

Complexity can be characterized by a certain degree of organization, structure, memory, and regularity [1]. The complexity should be zero in the extreme cases of complete randomness and perfect order. At a given distance from these extremes, a wide range of possible structures exists. The complexity measure does much more than satisfy the boundary conditions of vanishing in the high- and low-entropy limits. In particular, the maximum complexity occurs in the region between the system's perfectly ordered state and the perfectly disordered one.

The perfect crystal and the isolated ideal gas are two typical examples of systems with minimum and maximum entropy, respectively. However, they are also examples of simple models and therefore of systems with zero complexity, as the structure of the perfect crystal is completely described by minimal information (i.e., distances and symmetries that define the elementary cell) and the probability distribution for the accessible states is centered around a prevailing state of perfect symmetry. On the other hand, all the accessible states of the ideal gas occur with the same probability and can be described by a "simple" uniform distribution.

Statistical complexity is often characterized by the paradoxical situation of complicated dynamics generated from relatively simple systems. If the system itself is already involved enough and is constituted by many different parts, it clearly may support a rather complex dynamics, but perhaps without the emergence of typical characteristic patterns [3]. Therefore, a complex system does not necessarily generate a complex output. Statistical complexity is, therefore, related to hidden patterned structures which emerge from a system which itself can be much simpler than the dynamics it generates [3].

According to López-Ruiz, Mancini and Calbet [5], and using an oxymoron, an object, a procedure, or system is said to be complex when it does not exhibit patterns regarded as simple. It follows that a suitable complexity measure should vanish both for completely ordered and for completely random systems and cannot only rely on the concept of information (which is maximal and minimal for the systems mentioned above). A suitable measure of complexity can be defined as the product of a measure of information and a measure of disequilibrium, i.e., some kind of distance from the equiprobable distribution of the accessible states of a system. In this respect, Rosso and coworkers [6, 4] introduced an effective *Statistical Complexity Measure* (SCM) $\mathcal{C}$, that is able to detect essential details of the underlying dynamical processes.

Based on the seminal notion advanced by López-Ruiz *et al.* [5], this statistical complexity measure [6, 4] is defined through the functional product form

$$\mathcal{C}[P] = \mathcal{Q}[P, P_e] \cdot \mathcal{H}[P] \tag{8}$$

of the normalized Shannon Entropy $\mathcal{H}$, see Eq. (3), and the disequilibrium $\mathcal{Q}$ defined in terms of the Jensen-Shannon divergence $\mathcal{J}[P, P_e]$. That is,

$$\mathcal{Q}[P, P_e] = Q_0 \cdot \mathcal{J}[P, P_e] = Q_0 \cdot \{\mathcal{S}[(P + P_e)/2] - \mathcal{S}[P]/2 - \mathcal{S}[P_e]/2\}, \tag{9}$$

the above-mentioned Jensen-Shannon divergence and Q_0, a normalization constant such that $0 \leq \mathcal{Q} \leq 1$:

$$Q_0 = -2 \left\{ \frac{N+1}{N} \ln(N+1) - \ln(2N) + \ln N \right\}^{-1}, \tag{10}$$

are equal to the inverse of the maximum possible value of $\mathcal{J}[P, P_e]$. This value is obtained when one of the components of P, say p_m, is equal to one and the remaining p_j are zero.

Note that the above introduced SCM depends on two different probability distributions: one associated with the system under analysis, P, and the other the uniform distribution, P_e. Furthermore, it was shown that for a given value of $\mathcal{H}$, the range of possible $\mathcal{C}$ values varies between a minimum $\mathcal{C}_{\min}$ and a maximum $\mathcal{C}_{\max}$, restricting the possible values of the SCM [7]. Thus, it is clear that evaluating the statistical complexity measure provides important additional information.

In statistical mechanics, one is often interested in isolated systems characterized by an initial, arbitrary, and discrete probability distribution. Evolution towards equilibrium is usually the main goal. At equilibrium, we may suppose, without loss of generality, that this state is given by the equiprobable distribution $P_e = \{p_i = 1/N, \forall i = 1, \dots, N\}$. The temporal evolution of the above introduced Information Theory quantifiers, Shannon Entropy $\mathcal{H}$, statistical complexity $\mathcal{C}$ and Fisher information measure $\mathcal{F}$, can be analyzed using two-dimensional (2D) diagrams of the corresponding quantifiers versus time t.

Two information planes are defined: *a)* The *Entropy–Complexity plane*, $\mathcal{H} \times \mathcal{C}$, is a compact manifold spanning values of the normalized Shannon Entropy $\mathcal{H}$ and the statistical complexity $\mathcal{C}$ based only on global characteristics of the associated time series PDF (both quantities are defined in terms of Shannon entropies); while *b)* the *Shannon-Fisher plane*, $\mathcal{H} \times \mathcal{F}$, is a compact manifold spanning values of the normalized Shannon Entropy $\mathcal{H}$ and the Fisher Information measure $\mathcal{F}$ based on global and local characteristics of the PDF. In the case of $\mathcal{H} \times \mathcal{C}$ the variation range is $[0, 1] \times [\mathcal{C}_{\min}, \mathcal{C}_{\max}]$ (the minimum and maximum statistical complexity values for a given $\mathcal{H}$ value [7]), while in the causality plane $\mathcal{H} \times \mathcal{F}$ the range is $[0, 1] \times [0, 1]$.

References

[1] D. P. Feldman, C. S. McTague, and J. P. Crutchfield. The organization of intrinsic computation: Complexity-entropy diagrams and the diversity of natural information processing. *Chaos: An Interdisciplinary Journal of Nonlinear Science*, 18(4):043106, 2008.

[2] B. R. Frieden. *Science from Fisher information: a unification.* Cambridge University Press, 2004.

[3] H. Kantz, J. Kurths, and G. Mayer-Kress. *Nonlinear analysis of physiological data.* Springer, 1998.

[4] P. Lamberti, M. T. Martin, A. Plastino, and O. A. Rosso. Intensive entropic non-triviality measure. *Physica A: Statistical Mechanics and its Applications*, 334(1-2):119–131, 2004.

[5] R. Lopez-Ruiz, H. L. Mancini, and X. Calbet. A statistical measure of complexity. *Physics Letters A*, 209(5-6):321–326, 1995.

[6] M. T. Martin, A. Plastino, and O. A. Rosso. Statistical complexity and disequilibrium. *Physics Letters A*, 311(2-3):126–132, 2003.

[7] M. T. Martin, A. Plastino, and O. A. Rosso. Generalized statistical complexity measures: Geometrical and analytical properties. *Physica A: Statistical Mechanics and its Applications*, 369(2):439–462, 2006.

[8] L. Pardo, D. Morales, K. Ferentinos, and K. Zografos. Discretization problems on generalized entropies and r-divergences. *Kybernetika*, 30(4):445–460, 1994.

[9] M. RA Fisher. On the mathematical foundations of theoretical statistics. *Phil. Trans. R. Soc. Lond. A*, 222(594-604):309–368, 1922.

[10] P. Sánchez-Moreno, R. Yánez, and J. Dehesa. Discrete densities and Fisher information. In *Proceedings of the 14th International Conference on Difference Equations and Applications. Difference Equations and Applications. Istanbul, Turkey: Bahçesehir University Press*, pages 291–298, 2009.

[11] C. E. Shannon. A mathematical theory of communication. *Bell System Technical Journal*, 27:379–423, 1948.

[12] C. E. Shannon and W. Weaver. *The mathematical theory of communication*, volume 117. University of Illinois, 1949.

[13] K. Zografos, K. Ferentinos, and T. Papaioannou. Discrete approximations to the Csiszár, Renyi, and Fisher measures of information. *The Canadian Journal of Statistics/La Revue Canadienne de Statistique*, pages 355–366, 1986.
